# Novel directions of precision oncology: circulating microbial DNA emerging in cancer-microbiome areas

**DOI:** 10.1093/pcmedi/pbac005

**Published:** 2022-02-03

**Authors:** Liting You, Juan Zhou, Zhaodan Xin, J Spencer Hauck, Feifei Na, Jie Tang, Xiaohan Zhou, Zichen Lei, Binwu Ying

**Affiliations:** Department of Laboratory Medicine, West China Hospital, Sichuan University, Chengdu 610041, China; Department of Laboratory Medicine, West China Hospital, Sichuan University, Chengdu 610041, China; Department of Laboratory Medicine, West China Hospital, Sichuan University, Chengdu 610041, China; Department of Pathology, Duke University School of Medicine, Durham, NC 27710, USA; Department of Thoracic Cancer, West China Hospital, Sichuan University, Chengdu 610041, China; Department of Clinical Laboratory, Mianyang Central Hospital, School of Medicine, University of Electronic Science and Technology of China, Mianyang 621000,China; Department of Laboratory Medicine, West China Hospital, Sichuan University, Chengdu 610041, China; Department of Laboratory Medicine, West China Hospital, Sichuan University, Chengdu 610041, China; Department of Laboratory Medicine, West China Hospital, Sichuan University, Chengdu 610041, China

**Keywords:** circulating microbial DNA, liquid biopsy, cancer–microbiome–immunity, intra-tumor microbiome, cancer precision diagnosis and therapy

## Abstract

Microbiome research has extended into the cancer area in the past decades. Microbes can affect oncogenesis, progression, and treatment response through various mechanisms, including direct regulation and indirect impacts. Microbiota-associated detection methods and agents have been developed to facilitate cancer diagnosis and therapy. Additionally, the cancer microbiome has recently been redefined. The identification of intra-tumoral microbes and cancer-related circulating microbial DNA (cmDNA) has promoted novel research in the cancer–microbiome area. In this review, we define the human system of commensal microbes and the cancer microbiome from a brand-new perspective and emphasize the potential value of cmDNA as a promising biomarker in cancer liquid biopsy. We outline all existing studies on the relationship between cmDNA and cancer and the outlook for potential preclinical and clinical applications of cmDNA in cancer precision medicine, as well as critical problems to be overcome in this burgeoning field.

## Introduction

Genetic and environmental factors are considered to contribute to the initiation, progression, metastasis, variation, and evolution of malignancies.^1^, ^2^ In recent years, previously underestimated roles of the microbiome in cancer are being taken seriously as the microbiome shows great prospects in cancer prevention, diagnosis, and treatment.^3^, ^4^ Actually, the roles of ubiquitous microbes in cancers^5^ remain hidden in the biological black box, although some of the associations between microbes and tumors have been revealed in many reports on certain cancer–microbial pairs.^6–8^

**Figure 1.**
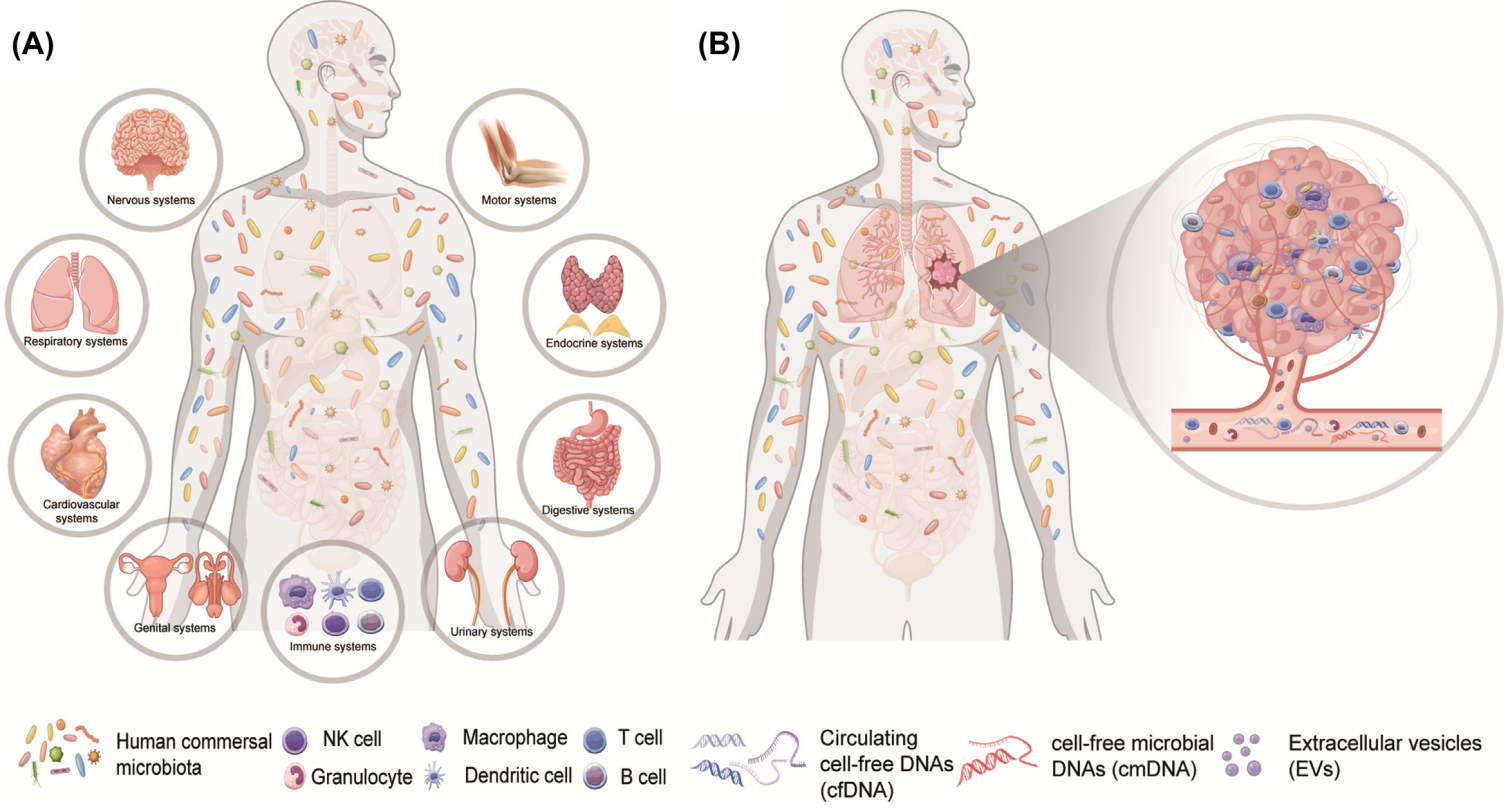
Definition of the human commensal microbial system and the microbiomes of cancer patients. (**A**) The commensal microbial system, as a novel separate organ system like the other nine systems in the human body, could interact with other human systems to maintain homeostasis; (**B**) the microbiomes of cancer patients include the intratumor microbiome, the circulating microbiome, and the microbiomes of other organs including the gut, lung, skin, oral cavity, etc.

Previous calculations showed that the microbes (mostly bacteria) coexisting in the human body represent 1%–3% of the overall body weight, ∼1–3 kg in a 70 kg adult, while the total number of bacteria in the body actually exceeds that of human cells.^9^, ^10^ In a symbiotic relationship, commensal microbiota can regulate many functions of human hosts.^11–17^ Typically, they play important roles in host immunomodulation,^18^, ^19^ such as in the formation and training of host immunity.^20^, ^21^ Perturbation of commensal microbiota may result in an impaired immune response to infectious and non-infectious factors. Consequently, the human microbiota are considered the largest immune organ of the human body. However, they do more than that. Other physiological and pathological functions, including cell proliferation and differentiation, circadian rhythmicity, metabolism, inflammation, tumor invasion and migration, as well as cancer treatment response, are in their repertoire.^18^ Hence, we propose that commensal microbes of the human body deserve to be considered as a separate organ system, named the commensal microbial system (Fig. 1A). Moreover, the underlying interactions of the microbial system and the other human systems deserve further exploration (Fig. 1A).

According to the studies over decades, microbes emerge and implement essential roles in tumor occurrence and development, such as human papillomavirus (HPV) in cervical cancer,^22^, ^23^ hepatitis B virus (HBV) in liver cancer,^24^ and helicobacter pylori (HP) in gastric cancer.^25^ These microbes and their nucleic acids and proteins can take effect by interacting with tumor signaling pathways.^26^, ^27^ Moreover, host immunity has been regarded as an important facet in the interaction of microbes and tumors.^18^ Gut microbiota contributes to shaping innate and adaptive immunity and is associated with the efficacy and adverse events of immune checkpoint inhibitors (ICIs) immunotherapy.^28^, ^29^ On the other hand, host immunity impacts on the interaction of microbes and cancer.^30^, ^31^ More recently, certain specific bacteria were identified in previously presumed aseptic tumor tissues and the concept of the intra-tumor microbiome (ITM) was established, which represents a novel direction in the cancer–microbiome–immunity areas.^32^ However, the source of intra-tumor microbes remains unclear; the roles that these microbes play in the development of cancer are still in discussion; and the potential application of ITM in cancer prevention, diagnosis, and treatment is even further down the road in this blossoming field.^33^ Excitingly, multiple studies have identified circulating microbial DNA (cmDNA) in plasma cell-free DNA (cfDNA) of cancer patients and healthy donors after ruling out possible contamination and have tried to utilize cmDNA in cancer diagnosis training.^34^, ^35^ Identification and studies of cmDNA propose a new direction for cancer liquid biopsies, which will promote the blossoming of cancer precision medicine.

The new view is that microbes or microbial nucleic acid not only exist in plasma and tumor cells of cancer patients but can also be detected in the peripheral blood of healthy individuals.^5^, ^36^ Furthermore, cmDNA can reflect the results of the interactions between microbiome, cancers and immunity, thus, comprehensive and detailed exploration of cmDNA could provide important clues for personalized diagnosis and treatment and facilitate the development of cancer precision medicine. The roles of the gut and intra-tumor microbes in malignancies have been well reviewed previously,^5^, ^6^, ^37^, ^38^ and the actions of the cancer–microbiome–immunity axis have also been summarized comprehensively.^39^ Therefore, in this review, we focus on the potential clinical applications of cmDNA. In summary, we specifically aim to: (i) provide a brief overview of the interactions between microbes, cancers, and immunity to provide a background for further cmDNA discussion; (ii) highlight the identification, source, and research prospects of cmDNA and summarize the exciting translational applications of cmDNA in precision medicine for tumor diagnosis, staging and typing, treatment and prognosis; (iii) dissect potential obstacles and critical problems of cmDNA that need to be addressed in future preclinical research and clinical laboratory applications; and (iv) propose the conceptual assumption of tumor microbial burden (TMbB) and list the research needed in this field in the future to promote the development of cancer precision medicine.

## Background of cmDNA liquid biopsy: microbiome–cancer–immunity studies

Long-term investigations have demonstrated that the human microbiome plays crucial roles in cancer susceptibility, development, and therapeutic response.^40–42^ Complex interactions occur among commensal microbes, cancers, and host immunity.^43–45^

### Composition of human microbiome for cancer patients

The broad-sense human microbiome should be defined as the collection of all microbes (bacteria, viruses, fungi, etc.) and their components (DNA, RNA, and proteins) located in every part of the human body, including gut, lung, oral, vaginal, peripheral blood microbiome, as well as any other microbiomes of parenchymal and interstitial tissues.^46^ For cancer patients, the definition should be established as the above concepts plus intra-tumor microbes. Recently, the intra-tumor microbiome is considered to be crucial (Fig. 1B) since Nejman *et al*. investigated >1500 human tumor tissues of seven different tumors plus adjacent normal tissues and demonstrated that living bacteria exist in tumor tissues.^33^ They found that different types of tumors and different cells in the same tumors have different bacterial species, DNAs, and RNAs.^33^ However, the source of the intratumor microbiota is rarely reported and summarized. We propose and discuss three possible source routes. (i) Exogenous sources, such as digestive tract, respiratory tract, and urogenital tract. Intra-tumoral microbes of intestinal, bronchial, and urogenital neoplasms may come from the corresponding organs connected to the outside, i.e., the digestive tract, respiratory tract, and urogenital tract. (ii) Inborn sources, such as parental heredity and intrauterine microbes. This suggests that the microbial fragments existing in the human genome or from normal or abnormal intrauterine flora may be present in the offspring and in some tumors. (iii) Peripheral blood sources, such as sepsis and bacteremia during infections. This means some microbial fragments may remain in blood or tissues after individuals recover from previous infections during the non-tumor-bearing or tumor-bearing stage. The specific mechanisms can be inferred as follows: first, numerous studies have shown that bacterial translocation may occur between the intestinal mucosa and sterile tissues and organs, as well as tumor tissues.^47^, ^48^ Septicemia or undetected bacteremia can also lead to the location of bacteria in the specific tissues and organs, or mucosal innate immune cells may engulf some bacteria and serve as a shield for them to be carried out to other organs through the lymphatic circulation. Additionally, horizontal gene transfer (HGT), also called lateral gene transfer, is a process by which genetic material is passed between microbes in a non-parent offspring fashion.^49^ Bacteria and virus DNA are transferable to human offspring if HGT occurs in human germ cells. Additionally, genetic material of the mitochondria is inherited materially to offspring in humans, while it is known that the mitochondria organelles evolved from natural archaea.^50^ Thus, we speculate that the microbiota exists and can change at any time and everywhere in the body and participates in tumorigenesis and development. In addition to intra-tumor microbes, circulating microbial molecules are another important part of the human microbiome (Fig. 1B). Emerging evidence demonstrated that cmDNA was significantly distinct between tumor patients and healthy individuals.^34^ We will provide an in-depth summary and discussion on the identification history, fragment source, basic research, and clinical application value of cmDNA later in this article.

### Studies of human microbiome with cancer susceptibility, cancer occurrence and development, cancer treatment efficacy and side-effects

**Figure 2.**
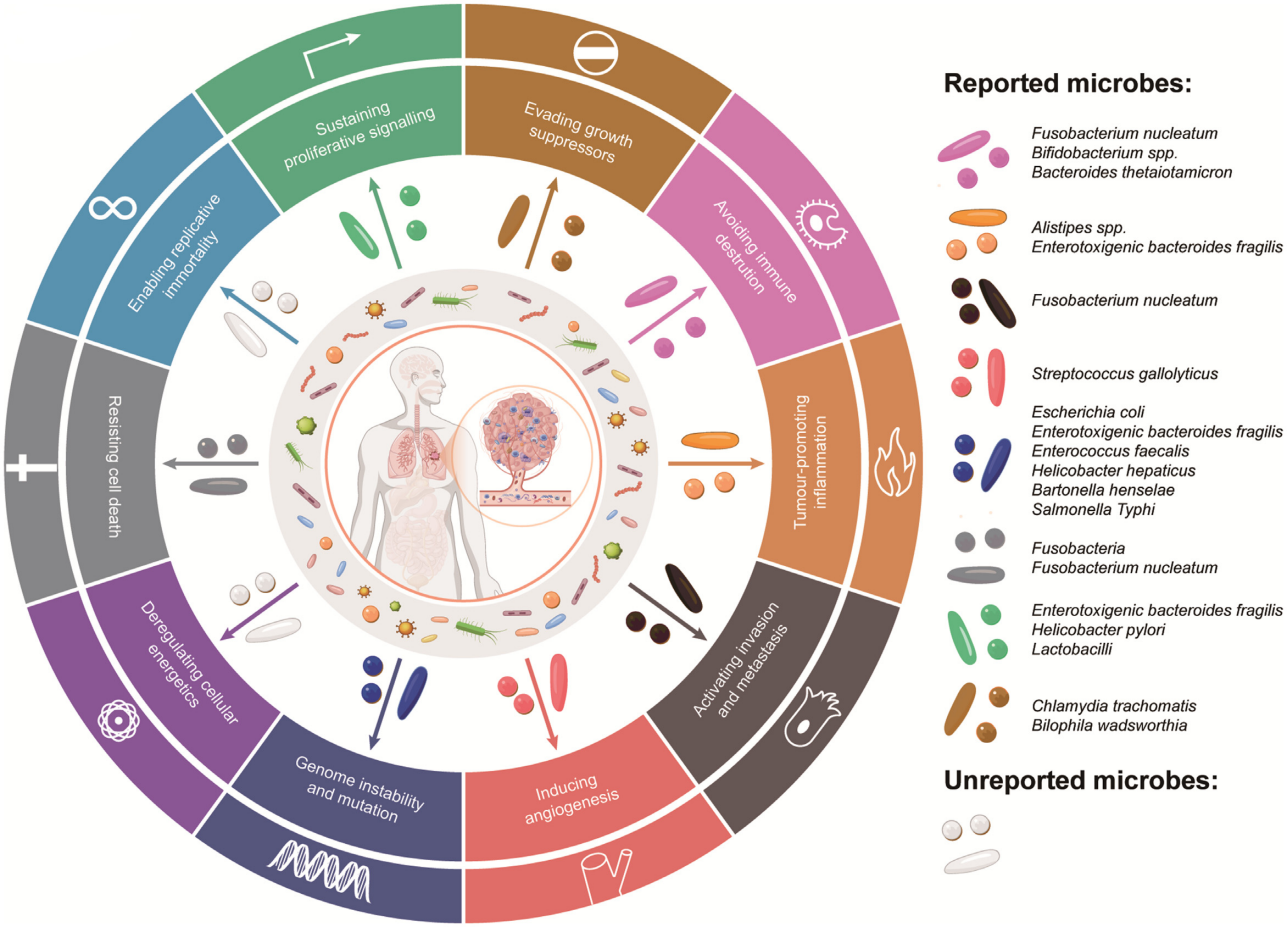
Interactions between microbes and cancer. Bacteria and viruses can implement important roles in tumorigenesis and progression through modulating the ten crucial hallmarks for cancer development.

It is estimated that 15.4% of human cancers are attributed to microbial infection at an early age.^51^ There are eleven microbes in the list of group 1 pathogenic carcinogens defined by the International Agency for Research on Cancer (IARC).^52^ Furthermore, some other microorganisms may be correlated to various tumor carcinogenesis as indicated from studies in animal models or clinical research (Table 1). Microbes can contribute to tumorigenesis, progression, and metastasis via a myriad of mechanisms and signaling pathways in the 10 hallmarks of cancer (Fig. 2).^26^, ^27^, ^53^ The linkage of carcinogenesis to viral infections is the most studied thus far.^54^, ^55^ Viral infection and genome integration can regulate almost all hallmarks of cancer to promote tumorigenesis, progression, and metastasis.^54^, ^56^ The roles of viruses in cancer initiation and progression are fairly complicated, in which virus subtypes and host states are both critical deciding factors for the fate of host cells. Many studies have explored the complex internal mechanisms.^23^, ^57–66^ For example, mechanisms of high-risk HPV (H-HPV) in cancer have been partly deciphered, including viral genome integration and E5, E6, and E7 effector proteins.^23^ H-HPV proteins can regulate intracellular signal transduction that promotes cancer progression,^23^ such as tumor-associated angiogenesis^57^ and immune-related molecular pathways.^58–60^

**Table 1.** Microorganisms that are reported to be associated with cancer genesis but not classified as group one pathogenic carcinogens.

Cancer	Microorganisms	References
Cholangiocarcinoma	Human Polyomavirus 6 (HPyV6), Human Polyomavirus 7 (HPyV7), Merkel cell polyomavirus (MCPyV)	^167^
Colorectal cancer	*Bacteroides fragilis, Escherichia coli, Enterococcus faecalis, Streptococcus gallolyticus, Parvimonas, Peptostreptococcus, Porphyromonas, Prevotella, Fusobacterium nucleatum*	^168^
Esophageal cancer	*Proteobacteria, Firmicutes, Bacteroidetes, Actinobacteria, Fusobacteria*	^169^
Gallbladder carcinoma	*Salmonella typhi*	^170^
Pancreatic cancer	*Neisseria elongate, Streptococcus mitis, Porphyromonas gingivalis, Bacteroidetes, Firmicutes, Proteobacteria, Synergistetes, Euryarchaeota, Saccharopolyspora, Pseudoxanthomona, Streptomyces*	^171^
Lung cancer	*Chlamydia pneumoniae*	^170^
Bladder cancer	*Staphylococcus aureus, Klebsiella spp., Proteus mirabilis, Acinetobacter, Fusobacteria*	^170^, ^172^, ^173^
Breast cancer	*Methylobacterium radiotolerans, Escherichia coli, Proteobacteria, Firmicutes, Actinobacteria, Brevundimonas, Mobiluncus, Candida, Geotrichum, Rhodotorula, Trichosporon, Epidermophyton, Trichophyton, Trichinella*	^174^, ^175^
Cervical cancer	*Lactobacillus, Atopobium vaginae, Dialister invisus, Finegoldia magna, Gardnerella vaginalis, Prevotella buccali, Prevotella timonensis*	^176^, ^177^
Endometrial cancer	*Atopobium vaginae, Porphyromonas sp*.	^178^
Ovarian cancer	*Proteobacteria, Firmicutes, Brucella, Chlamydia, Mycoplasma, M. genitalium, C. trachomatis, Neisseria gonorrhoeae*	^179–181^
Oral squamous cell carcinoma	*Porphyromonas gingivalis, Fusobacterium nucleatum*	^182^, ^183^
Prostate cancer	*C. trachomatis, N. gonorrhoeae*	^184^

Oncogenic mechanisms linked to bacterial infection are complex multi-step biological processes and involve the alteration of multiple signal transduction pathways, which are yet to be deciphered.^67^ It is generally assumed that bacteria-mediated inflammation responses are crucial links of malignant transformation.^68^ During inflammation processes, bacteria can escape immune defenses and survive via multiple mechanisms, including cellular antigen modification and variation, secretion of cytolytic protein toxins to eliminate immune cells, and antigen mimicry.^69^, ^70^ Long-term recurrent chronic inflammation stimulates cell proliferation to induce more base pair mismatches, insertion/deletion mutations, and the consumption of DNA mismatch repair (MMR) proteins, which increase the malignant transformation potential of host cells.^71^, ^72^ On the other hand, bacteria effectors and toxic proteins can regulate the 10 hallmarks of cancer through activation of STAT3, MAPK, and AKT oncogenic pathways^73^, ^74^ and inhibition of the P53 tumor-suppressor pathway.^68^ In addition, HGT could be another important mechanism. HGT of bacterial DNA was rarely reported until recently. Schroder *et al*. reported that the bacterial pathogen *Bartonella henselae* can transfer DNA into the genome of the human endothelial cell line EA.hy926.^75^ Riley *et al*. demonstrated that bacterial DNA integrations in human cells were more common in tumor cells and *Pseudomonas*-like DNAs integrate into the sites of four oncogenes to induce gastric adenocarcinoma.^76^ Multiple teams proposed that genome integration of bacterial DNA is one of the most important oncogenic mechanisms.^77–79^

Aside from cancer occurrence and progression, microbes also play crucial roles in modulating the response to cancer treatment.^40^, ^80^, ^81^ For instance, intestinal *Bifidobacterium pseudolongum* can enhance the efficacy of ICIs immunotherapy and its inosine metabolites modulated the response to ICIs.^82^, ^83^ It was reported that gut *Bacteroides ovatus* and *Bacteroides xylanisolvens* could increase lung cancer response to erlotinib, a kind of molecular targeted drug.^84^ Additionally, the latest reports claimed that intra-tumor microbes of pancreatic adenocarcinoma (PAAD) tissues, containing bacteria predominately from the *Enterobacteriaceae* and *Pseudomonadaceae* families, can modulate the resistance of PAAD patients to gemcitabine.^32^ Intra-tumor *Fusobacterium nucleatum* and *Bifidobacterium* were found to be related to the response to colorectal cancer chemotherapy.^85^, ^86^ In addition to bacteria, viruses are also closely related to tumor treatment outcomes.^87–89^ For example, a Hodgkin's lymphoma patient developed significant tumor remission after COVID-19 infection without any anti-tumor treatment.^90^ Tumor mycoplasma infection can reduce the efficacy of the anti-tumor drug gemcitabine via mycoplasma-encoded deaminase.^91^, ^92^ Presently, microbe-based therapies, including oral prebiotics or probiotics, fecal microbiota transplantations (FMT), or dietary interventions, are being examined as adjuvant strategies of cancer treatment in clinical trials (Table 2). Besides, there are many clinical trials about the safety and efficacy of oncolytic viruses in cancer treatment that are detailed in Table 2.^93^ Commensal microbiota can also impact on the adverse effects of cancer therapy, including ICIs immunotherapy,^94–96^ chemotherapy,^44^ and radiotherapy.^80^, ^97^ The predictive performance of the gut microbiome for radiotherapy-related toxicity is being evaluated by a clinical trial, NCT04638049.^98^ A clinical trial, NCT03516461, is being conducted by Zhang*et al*. to assess the efficacy and safety of FMT for radiation-induced enteritis of abdominal radiotherapy.^99^

**Table 2.** Active clinical trials of bacterial agents and oncolytic viruses applying to cancer therapy and adjuvant therapy.

Bacteria-related agents or methods for cancer therapy and adjuvant therapy			
Interventions	NCT number	Cancer types	Purpose
Fecal microbiota transplantation (FMT)	NCT03819296	Melanoma or genitourinary cancer	Effectiveness in medication-induced gastrointestinal complications
	NCT04163289	Renal cell carcinoma	Reducing the occurrence of immunotherapy-related adverse events (irAEs)
	NCT03772899	Melanoma	Safety and efficacy in immunotherapy patients
Mixed prebiotics or probiotics	NCT03773003	Various tumors	Investigating the possible benefit for cancer-related fatigue
	NCT03552458	Head-and-neck cancer	Efficacy in oral mucositis of patients undergoing head and neck radiotherapy
	NCT04021589	Metastatic colorectal cancer	Efficacy in cancer patients with chemotherapy
	NCT03870607	Anal cancer squamous cell	Efficacy in increasing the effectiveness of conventional treatment
Microbial ecosystem therapeutics strains (MET-4)	NCT03686202	Solid tumors	Effectiveness in cancer patients with immunotherapy
	NCT03838601	Oropharyngeal squamous cell carcinoma	Effectiveness in cancer patients with chemoradiotherapy
MRx0518, a strain of Enterococcus species	NCT03934827	Solid tumors	Examining anti-cancer and immune system modulating actions
	NCT04193904	Resectable pancreatic cancer	Evaluating safety and efficacy in preoperative hypo-fractionated radiation
*Clostridium butyricum* CBM588	NCT03922035	Hematopoietic and lymphoid cell neoplasm	Safety and efficacy in patients after hematopoietic stem cell transplant
Microbiome-based personalized diet intervention	NCT04079270	Breast cancer	Effectiveness in breast cancer patients with adjuvant endocrine treatment
Adenovirus	NCT02705196, NCT03916510, NCT03740256, NCT03618953, NCT04695327, NCT02798406, NCT04217473, NCT03190824, NCT03225989, NCT03714334, NCT03852511, NCT04053283, NCT04097002, NCT03896568, NCT03072134, NCT04685499, NCT03178032, NCT03003676	Pancreatic cancer, locally advanced rectal cancer, solid tumors, HPV-associated cancers, brain cancer, metastatic melanoma, ovarian cancer, biliary carcinoma, colorectal cancer, glioblastoma, epithelial tumors, prostate cancer, glioma, Head and neck squamous cell carcinoma, diffuse pontine gliomas etc.	To explore the safety, tolerance, and efficacy of oncolytic virus agents as a therapeutic or adjuvant therapeutic strategy in treating various cancer patients
Herpes simplex virus	NCT03004183, NCT04637698, NCT03866525, NCT03252808, NCT04386967, NCT02779855, NCT04185311, NCT03152318, NCT04735978, NCT04349436, NCT03911388, NCT03767348, NCT02457845, NCT04616443, NCT04348916, NCT02062827, NCT03657576	Metastatic triple negative breast cancer and non-small cell lung cancer, pancreatic cancer, solid tumors, breast cancer, glioma, cutaneous squamous cell carcinoma, brain tumors, melanoma, glioblastoma	
Vaccinia virus	NCT03206073, NCT03954067, NCT03294486, NCT02977156, NCT04301011, NCT02759588, NCT04725331, NCT04226066, NCT03294083	Colorectal cancer, solid tumors, glioblastoma, brain cancer, ovarian cancer, renal cell carcinoma	
Other types	NCT04787003, NCT04673942, NCT04445844, NCT03889275, NCT03605719, NCT04215146, NCT04102618, NCT04521764, NCT01846091, NCT02068794, NCT02364713, NCT03043391	Solid tumors, breast cancer, recurrent plasma cell myeloma, head and neck squamous cell carcinoma, recurrent ovarian, primary peritoneal or fallopian tube cancer, fallopian, or peritoneal cancer, children glioma	

### Human microbiome and host immunity

**Figure 3.**
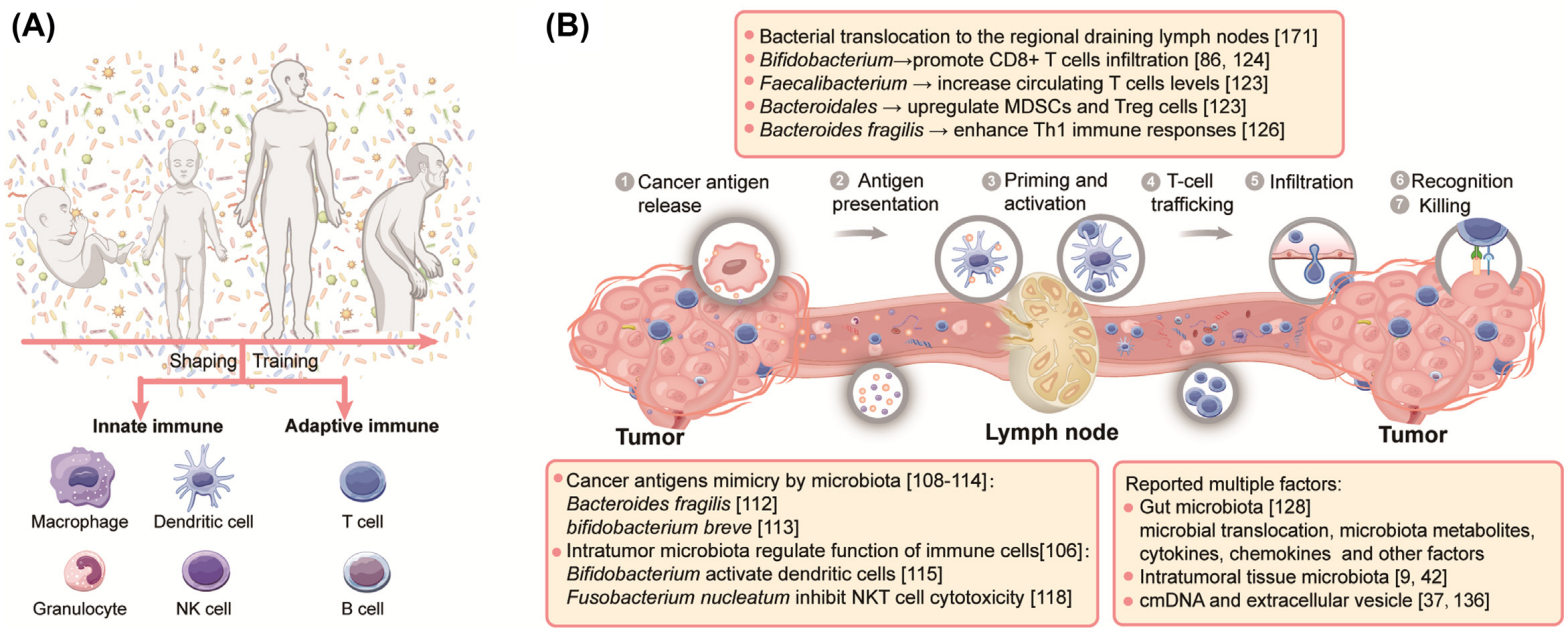
Interactions between the human microbiome and the immune system. (**A**) An individual is exposed to enormous communities of microbes throughout his or her life, and these microbes could play fundamental roles in the induction, regulation, training, and education of host immune function, including innate immunity and adaptive immunity. In reverse, the host immune system could modify the composition of microbial communities. (**B**) Microbes at different sites in cancer patients can regulate the anti-tumor immune response by modulating events in the seven-steps of the cancer–immunity cycle. MDSC: myeloid-derived suppressor cell, NKT: natural killer T cell.

Human resident microbes are influenced by intrinsic and environmental factors during an individual's lifetime which shape the host's immune characteristics.^100^, ^101^ The enormous communities of commensal microbes play a fundamental role in the induction, regulation, training, and education of host immune function,^19^, ^102^ including innate immunity and adaptive immunity (Fig. 3A). The co-evolution and mutual adaptation between human immunity and commensal microbiota impact the reactivity of immune systems to new-emerging malignant cells.^103^ Therefore, the effectiveness of the anti-tumor immune response also varies during oncogenesis and development due to the action of microbiota, which is closely related to the efficacy of cancer therapy,^91^, ^104^ as has been mentioned in previous reviews.^39^

After cancer occurrence, human commensal microbiota at different sites of cancer patients can regulate the anti-tumor immune response by modulating the seven-step events in the “cancer–immunity cycle” (Fig. 3B). The first step in an effective anti-tumor immune response is the release and recognition of cancer antigens. Previous studies have reported that cancer antigen molecular mimicry mediated by human microbiota is one of the most important mechanisms by which microbes participate in an anti-tumor immune response.^105–111^ For example, because of the homology and cross-reactivity between *Bacteroides fragilis* and tumor antigens, *Bacteroides fragilis*-specific T cells could restore the therapeutic activity of ICIs immunotherapy in mice.^109^ Aside from tumor antigen mimicry, commensal microbes originating in the gut,^112–116^ tumor tissues,^86^ and other sites of human bodies^117–119^ can regulate the function of antigen presentation cells and other immune cells.^103^, ^120^

After cancer antigen presentation, T cells are activated and translocate from the lymphatic system and infiltrate into tumor sites. The human microbiota system could impose crucial actions on these steps (Fig. 3B).^121^ It has been reported that gut *bifidobacterium, faecalibacterium, B. fragilis* could increase T cells levels and enhance T cells tumor infiltration to promote an anti-cancer immune response.^83^, ^122–125^ Human microbiota can also regulate the function of immune cells and stimulate the secretion of cytokines and chemokines.^126–131^ For example, it is reported that intra-tumor *salmonella enterica serovar typhimurium* can colonize into tumor by vascular disruption and increase tumor necrosis factor-alpha (TNF-α) secretion.^132^ Besides, intratumoral bacterial lipopolysaccharide, lipoteichoic acid, and 16S rRNA/DNA may modulate immune cells of the TME to influence anti-cancer effects.^33^ Third, the potential roles of microbial DNA circulating in peripheral blood or extracellular vesicles remain undiscovered. cmDNA as a kind of cell-free DNA (cfDNA) may be just a transient passenger, but in some situations it plays substantive roles, such as immunomodulatory actions.^133^ TLR9, PRR, and cGAS-STING signals are all identified DNA-stimulated immune response pathways.^134^ In fact, the underlying phenotypes and mechanisms of cmDNA need more exploration.

## Novel directions of cancer liquid biopsy: circulating cell-free microbial DNA

Peripheral blood travels throughout the body of cancer patients and carries certain molecules from the tissues, including messages of the cancer microbiome such as DNA, RNA, and metabolites.^36^ Microbes and viruses from the whole body of a cancer patient were demonstrated to be strongly involved in cancer development, metastasis, cancer-immune regulation, cancer treatment response, and clinical outcomes.^135^ As a result, these cancer regulatory molecules from the cancer microbiome theoretically enter into blood and could be detected through some methods with sufficient sensitivity, e.g. cmDNA is one of the the most important circulating microbial laboratory items. Circulating tumor DNA (ctDNA) and circulating tumor cells (CTCs) have become excellent examples of circulating biomarkers of solid tumors in clinical diagnosis and therapy in recent years. Reasonably, cmDNA has similar potential for clinical application in cancer liquid biopsies.^136^

### Source of cmDNA in cancer patients

**Figure 4.**
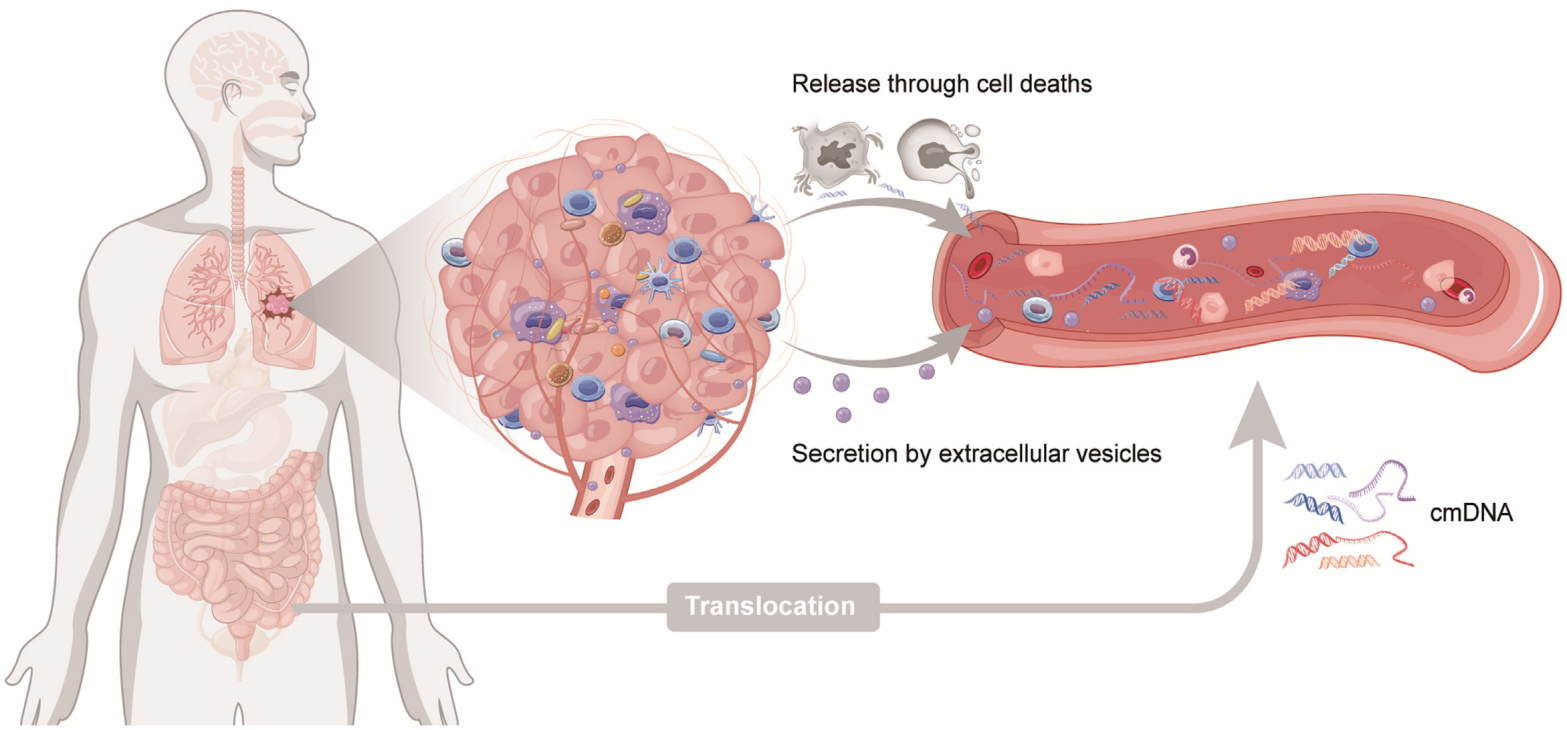
Hypothesized mechanisms by which microbial DNA enters the peripheral circulation. The release of tumor microbial DNA following cell deaths in cancer tissues, the secretion of vesicles containing microbial DNA, and translocation of intestinal microbial DNA are all the potential sources of cmDNA.

Referring to the previously described knowledge about the source of cfDNA, here, we discuss the sources of cmDNA from cancer patients for the first time as follows (Fig. 4): (i) passive release of endogenous microbial DNA after cell death, including apoptosis, necrosis, pyroptosis, and ferroptosis of cancer cells, immune cells, and any other cells; (ii) active secretion of cells, including eukaryocytes from cancer patients and prokaryocytes from commensal bacteria; (iii) microbe translocation and DNA release; and (iv) partially impaired immune clearance of cmDNA.^137^, ^138^ During the process of cancer development, tumor cells grow and proliferate rapidly and compete with each other for relatively inadequate nutrients. Consequently, large number of tumor cells that die from apoptosis or necrosis release nucleic acids and other molecules. Besides, pyroptosis and ferroptosis are also closely associated with cancer development.^139–141^ Various forms of cellular deaths of tumor cells may lead to the release of microbial DNA, RNA, and protein which have been demonstrated to exist in tumor tissues in lung, esophageal, colorectal, and pancreatic cancers.^5^ Interestingly, microbial DNA could be secreted into blood in the form of extracellular vesicles (EVs). The pathway of viral packaging, biogenesis, and transmission can overlap with secretion and delivery of EVs; EVs and virus particles share common structure, size, and uptake process.^142^ Several studies have reported that EVs carry viral genetic materials and envelope proteins.^143^ A study of hepatocellular cancer exosomes conducted by Yang *et al*. found that plasma exosomes containing HBV DNA and proteins can transfer nucleic acid fragments into other cells including hepatocellular cells and immune cells, and they demonstrated that HBV-positive exosomes induced dysfunction of natural killer cells.^144^, ^145^ Several studies also reported that Hepatitis C Virus (HCV) RNA existed in plasma exosomes that can modulate immune function by transmitting HCV.^146^, ^147^ Prokaryotes are also able to generate EVs,^148^ but the EVs from bacteria and viruses can trigger a strong immune reaction and are often cleared rapidly. Microbial DNA and other components need to be wrapped by host-source EVs to escape immune clearance. In theory, microbial DNA in EVs reflects the composition of the human microbiome and cmDNA in blood EVs may serve as a promising biomarker of cancer.^138^, ^149^ Furthermore, microbiota from gut, lung and other organs can translocate into the whole body via blood circulation without causing a systemic infection.^150^ Given that Xiao *et al*. have observed that the source of cmDNA was mostly from gastrointestinal genera as well as the oral tract microbiome,^182^ we hypothesize that part of cmDNA in plasma could derive from translocation of gut microbiota as well as the microbiome within tumors which spread into the bloodstream. However, partial or total impairment of the host immune clearance function is the decisive condition for cmDNA to survive.^137^ Cancer patients are often in an immune-compromised status and their immune function is often impaired, which allows cmDNA to survive and be detected in tumors and blood.

### The potential clinical applications of cmDNA in cancer diagnosis, staging, and prognosis monitoring

Remarkably, emerging evidence in recent years has demonstrated that highly divergent cmDNA showed promising diagnostic and prognostic implications across diverse cancer types (Table 3). Huang *et al*.^151^ and Cho *et al*.^152^ first noticed the potential links between cmDNA and cancer through examining the diagnostic performance of cmDNA in early-onset breast cancer (EOBC) and hepatocellular carcinoma (HCC), respectively.^151^ Though only five samples were analyzed, Huang *et al*. found significant differences in bacterial species between breast cancer patients and healthy individuals. EOBC patients had high titers of cmDNA derived from *Pseudomonas* or *Sphingomonas spp*., while cmDNA of healthy females was derived from *Acinetobacter spp*. These results hint that cmDNA from different species of bacteria could serve as indicators in cancer diagnosis. Similarly, Cho *et al*.^152^ evaluated the diagnostic performance of cmDNA in HCC. Through blood metagenomic analyses of 79 HCC patients, 83 cirrhosis patients, and 201 healthy controls, they observed that microbial diversity was reduced in HCC, suggesting that cmDNA characteristics could distinguish HCC patients from others. Next, they constructed a diagnostic model containing 5-genera of microbes showing area under the ROC curve (AUC) values of 0.879 (0.729 sensitivity; 0.850 specificity; 0.816 accuracy) in the train set and 0.875 (0.756 sensitivity; 0.797 specificity; 0.798 accuracy) in the test set, indicating the great potential of cmDNA in HCC diagnosis. Besides, Dong *et al*.^153^ found cmDNA from *acinetobacter, bacteroides*, and *haemophilus parainfluenzae* was enriched in the serum of gastric cancer patients.

**Table 3.** Reported studies evaluating the diagnostic and staging performance of cmDNA in different types of cancers.

Cancer type	cmDNA signatures	Cohort	Functions of cmDNA
Prostate cancer, lung cancer, and melanoma^34^	*Aliivibrio* genus using both Kraken and SHOGUN-derived taxonomy assignments	Non-cancer, HIV-, healthy controls (*n* = 69) and 100 patients from three types of high-grade (stage III–IV) cancers: prostate cancer (*n* = 59); lung cancer (n = 25) and melanoma (*n* = 16)	Diagnostic performance in distinguishing cancers from healthy patients
Early-onset breast cancer^151^	Early-onset breast cancer patients had high titers of cmDNA derived from *Pseudomonas* or *Sphingomonas spp*.	Early-onset breast cancer patients (*n* = 3) and healthy females (*n* = 2)	Potential diagnostic and prognostic value of the cmDNA profiles
Hepatocellular carcinoma^152^	5-Genera microbiome signature (*Pseudomonas, Streptococcus, Staphylococcus, Bifidobacterium*, and *Trabulsiella*)	Patients with HCC (*n* = 79) and cirrhosis (*n* = 83), and matching healthy controls (*n* = 201)	Potential diagnostic value in distinguishing HCCs from healthy controls
Gastric cancer^153^	Enriched *acinetobacter, bacteroides*, and *hemophilus parainfluenzae* in gastric cancer	Gastric cancer (*n* = 71), atypical hyperplasia (*n* = 6), chronic gastritis (*n* = 11), and healthy controls (*n* = 13)	Potential value of cmDNA in diagnosis, progress evaluation, and prognosis prediction of gastric cancer
Colorectal cancer (CRC)^154^	28 Microbial species (e.g. *Eubacterium rectale, Bifidobacterium adolescentis, Ruminococcus torques, Roseburia intestinalis*, and *Propionibacterium freudenreichii*)	CRC patients (*n* = 25), colorectal adenoma (CRA) patients (*n* = 10) and healthy controls (*n* = 22)	Potential non-invasive biomarkers in early diagnosis of CRC
Colorectal cancer^155^	DNA coding for 16S rRNA, *β-*galactosidase of *E. coli*, glutamine synthase of *B. fragilis*, DNA coding for 5.8S rRNA of *C. albicans*	CRC patients (*n* = 397) and healthy controls (*n* = 32)	Promising prognostic (progression free survival (PFS) and overall survival (OS)) biomarkers of CRC patients
Melanoma^156^	A significant differential abundance of *Castellaniella* genus between melanoma and healthy control plasma samples	Stage IV melanoma patients (*n* = 15) and healthy controls (*n* = 15)	cmDNA can serve as a potential biomarker after removal of contamination

After that, Poore *et al*.^34^ re-analyzed sequencing data of 18 116 tumor samples across 10 481 patients and 33 cancer types from The Cancer Genome Atlas (TCGA) database. By controlling possible contamination and utilizing machine learning, they identified microbial signatures discriminating cancer types after several rounds of modeling. As the contamination control is one of the most crucial links, they identified and excluded external contaminations by using the method of sample analyte concentrations described in previous reports and the method of identifying a “blacklist” of microbes from reagents of the same manufacturers and combining the method of manually reviewed the literatures. In addition, internal contaminations during sequencing or data processing were identified by conventional identification methods and Bayesian analyses. Via the blood sequencing data mining, they found cmDNA patterns performed well at distinguishing early-stage tumors from normal tissue. Then they validated the performance of cmDNA in a separate cohort (prostate cancers vs. healthy individuals: AUC 0.9477; lung cancers vs. healthy individuals: AUC 0.9716). The study demonstrated that cmDNA has great feasibility and generalizability as a promising biomarker for cancer liquid biopsy in clinical settings. Following the above studies, Xiao *et al*.^154^ further validated the diagnostic value of cmDNA in colorectal cancer (CRC) by performing whole genome sequencing (WGS) on plasma samples of 25 CRC patients, 10 colorectal adenoma (CRA) patients, and 22 healthy controls. They observed that 127 species showed significant differences between CRC patients and healthy controls. Then they used a random forest model to furtherly identify 28 microbial species, and to distinguish CRC/CRA from healthy controls. Additionally, the diagnostic performance of these 28 microbial species was validated via 1X WGS in an additional cohort. Furthermore, Messaritakis *et al*.^155^ reported the clinical value of cmDNA in predicting outcomes and monitoring treatment efficacy of CRC patients. They found cmDNA, including 16S rDNA, β-galactosidase of *Escherichia coli*, glutamine synthase of *B. fragilis*, and 5.8S rDNA, was correlated with disease progression and survival of CRC patients. The potential value of cmDNA was also reported by Zozaya-Valdés *et al*.^156^ in melanoma, although they indicated that the problem of contamination should be addressed.

**Figure 5.**
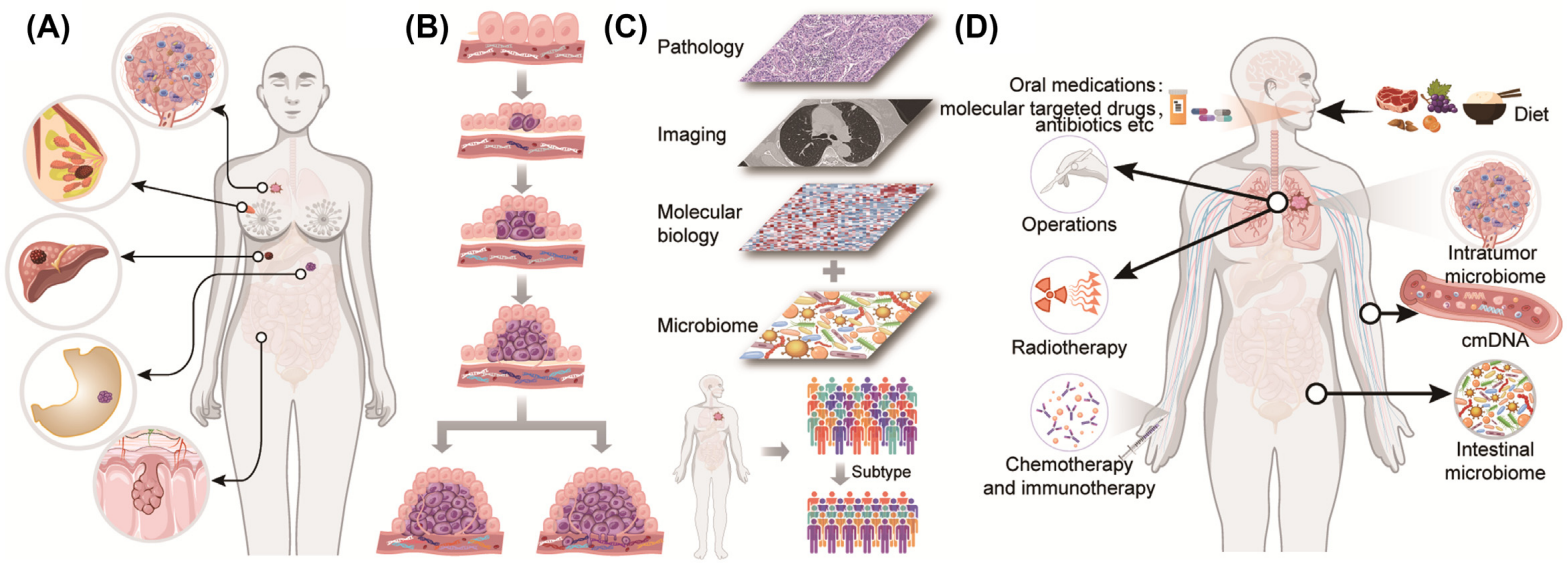
The underlying clinical value and research directions of cmDNA in cancer patients. (**A**) Reported cancers in which cmDNA showed excellent diagnostic performance; (**B**) cmDNA has been revealed to have great value in cancer staging and early diagnosis; (**C**) a combination of pathological, imaging, and molecular characteristics with microbiome profiles of cancer patients may contribute to the differential diagnosis of tumor subtypes, providing more clues for clinical diagnosis and treatment; (**D**) intestinal microbes of cancer patients, intra-tumor microbes, and cmDNA are influenced by diet and drugs, and are closely associated with efficacy and adverse events of anti-tumor drugs as well as patient outcomes.

Above all, cmDNA showed great performance in precise diagnosis and staging of malignancies, including lung cancer, prostate cancer, CRC, gastric cancer, EOBC, and HCC (Fig. 5A), which demonstrated that the diagnostic methods targeting cmDNA possess promising clinical potential. According to these studies, the levels and species of cmDNA in cancer patients could not only distinguish cancer patients from healthy controls but could successfully differentiate early-stage from late-stage patients, suggesting underlying value in cancer staging and early diagnosis (Fig. 5B).^157^ Moreover, some researchers have identified the essential microbiota for the distinction of different tumor subtypes on the basis of the optimal features produced from diverse microbiome computational methods, providing the possibility of cmDNA serving as a cancer-subtype biomarker.^158–160^ In present clinical applications and explorative studies, the differentiation of tumor subtypes is mainly guided by a combination of pathology, imaging, and molecular biology.^161^ Actually, the heterogeneity of cancers far exceeds the current criteria for dividing tumor subtypes. Immune phenotypes of the tumor microenvironment have been shown to be novel and effective methods for tumor typing in recent reports. Intra-tumoral microbes and microbial components show regular and statistical differences in different types of immune cells of the microenvironment, which hints that intra-tumoral microbes are associated with tumor immunophenotype. As such, various signatures of cmDNA may represent different cancer subtypes. Therefore, the inclusion of patients’ microbiome profiles may bring unexpected assistance in accurate diagnosis and prognosis for cancer subtypes (Fig. 5C). cmDNA has great advantages for clinical application, including non-invasive biopsy, and would reflect therapeutic efficacy more easily with prospects for further applications (Fig. 5D). Besides, with cancer progression, the abundance and the detectable rates of cmDNA would probably be higher, indicating a worse prognosis for patients (Fig. 5D). At present, microbial-based therapy of cancer has been one of the emerging cancer treatment modalities during the past few years, yet cancer therapy strategies based on cmDNA have not been explored.^162^ The use of specific types of microorganisms as cancer treatment is expected to stimulate the immune system for selective elimination of cancer cells and could lead to promising results.^163^ Targeting microbiota could be used as adjuvant treatments to improve therapeutic efficiency and reduce related toxicity. After in-depth exploration of the internal roles of cmDNA in tumorigenesis and progression, cmDNA-based cancer therapy combined with exosomes and liposome technologies could also serve as an effective strategy for cancer treatment in the future. In conclusion, cmDNA was found to show promising potential in diagnostic, subtyping, therapeutic response, and prognostic prediction biomarkers for cancer patients (Fig. 5). However, additional studies with larger cohorts and functional mechanisms are warranted to validate this hypothesis. In a word, exploration and application of cmDNA represent novel directions for cancer precision medicine.

## Problems to be overcome in preclinical research and clinical application of cmDNA

In spite of the great potential of cmDNA, numerous problems remain to be addressed in this growing field. We will group the potential problems into the following three aspects: preanalytical, analytical, and postanalytical phases.

### Preanalytical problems

Before cmDNA detection becomes a routine test in the clinical laboratory, its theoretical reliability and reasonability in cancer diagnosis or prognosis monitoring still need to be confirmed in more large-sample-size and multicentric studies, and it must be examined in real clinical settings with more complex subpopulations, including different gender, age, ethnicity, racial background, and effects induced by many benign diseases. Most notably, traditional theories suggest that only tumors specifically associated with microbial infection could be diagnosed according to cmDNA, such as liver cancer, gastric cancer, etc. Actually, for those tumors in which microbial infection is not a necessary condition, such as lung cancer, microbiota signatures could not only be used for cancer diagnosis, but also show some potential in cancer staging.^34^ The underlying mechanisms of intra-tumoral microbes and cmDNA in cancer initiation and progression remain unclear. We hold the view that it is not a simple cause-and-effect relationship between microbes and tumors but involves cross-interactions that occur both before and after tumorigenesis and during cancer progression. Although cmDNA is not as specific as CTCs for cancer diagnosis, the potential should not be underestimated. Based on the present evidence, the preanalytical problems that need to be considered in sample collection and pre-handling can be summarized as four “Ws” and an “H”, who, when, what, why, and how. First, “who” refers to the intended population of cmDNA detection. In studies screening tumor-related cmDNA for cancer diagnosis, cancer patients who present with an infection should be excluded. In addition, patients with benign disease should be included as controls to reflect the real application value of cmDNA detection, and the impact of subpopulations like gender and age on the clinical application of cmDNA should also be considered. Second, “when” refers to the time point of sample collection and minimum transport time after collection. Sample collection during microbial infection or antibiotic administration (at least 1 month apart) should be avoided. As a newly emerged liquid biopsy biomarker, cmDNA needs to be evaluated in different clinical situations, including tumor differential diagnosis, treatment guidance, postoperative monitoring, as well as the follow-up inspection after chemotherapy and radiotherapy, which may indicate different clinical problems. The timepoint of sample collection, days or months after treatment, will also matter. Besides, the minimum transport time for samples depends on various factors,^133^ including the use of a cfDNA preservation solution. Third, “what” refers to the cmDNA detection methods to be implemented. In the research phase, metagenomic next-generation sequencing (mNGS), 16s DNA sequencing, and targeted mNGS were usually considered to screen related cmDNA, whereas in the phase of clinical application, simple, fast, and low-cost methods were usually adopted, such as targeted mNGS, qPCR, and droplet digital (ddPCR). The last W, “why”, refers to different indications for clinical applications of cmDNA testing, including early screening for cancer, diagnosis, therapeutic efficacy monitoring, and prognosis evaluation. Finally, the “H”, "how", contains all the details of cmDNA detection techniques and procedures in a clinical laboratory, including sample volume, selection of anticoagulant, sample storage conditions, sample standard operation procedure, and all quality control (QC) measurements. Among them, QC measurements are the most important. For example, the disturbance of numerious cfDNA from leukocytes and the hindrance of other blood components, such as cell debris of hemolysis, should be eliminated to improve the sensitivity of cmDNA detection. Furthermore, microbial contamination control should be taken into consideration during sample collection and processing, e.g. cutaneous and environmental contamination should be considered. The use of blank environmental controls is one of the most effective strategies of microbial contamination control. In summary, corresponding strategies for all of the above problems should be well prepared before cmDNA detection and analysis.

### Analytical problems

Preclinical research and laboratory application of cmDNA in cancer diagnosis, cancer stage, and prognosis involve multi-step procedures. Taking the mNGS method as an example, this procedure involves the extraction of plasma cfDNA, preparation before sequencing, mNGS, and bioinformatic analyses. First, specificity and sensitivity are significantly crucial in a routine laboratory test. The specificity of cmDNA detection depends on selection of cmDNA panels of the corresponding microbial species in the study. The sensitivity of cmDNA detection depends on various factors, including plasma cmDNA extraction efficiency and detectable rate. Before the cfDNA extraction step, the cmDNA capture efficiency of various cfDNA extraction kits should be evaluated because cmDNA is more fragmented than other cfDNA.^164^ In order to improve the detectable rate of cmDNA, an mNGS method with high coverage and resolution is preferable in the research phase.^34^, ^35^ For the clinical application phase, simpler methods like PCR, more sensitive ddPCR, or lower-cost 16S sequencing methods are preferable.^165^ For mNGS, improvements in the methods of DNA library construction, such as the ssDNA method,^166^ and an increase of sequencing depth, such as a depth of (25–30)× used in the study by Xiao *et al*.,^35^ could improve the sensitivity of cmDNA detection. PCR and ddPCR methods are highly sensitive, and the design of primers and probes, as well as use of the multiplex PCR method, can improve the specificity of amplification and detection. Other strategies include the combined application of cmDNA detection with other clinical laboratory inspection items for cancers, such as ctDNA detection and analyses. Microbial contamination-induced false positivity is another problem of concern in cmDNA detection and analysis. Microbes are everywhere, in human bodies, in the environment, instruments, consumables, and reagents, consequently, microbial contamination can occur from sample processing to testing. It is critically important to test negative controls accompanied by the tested samples to remove possible contaminations. Qualified negative controls usually contain mixtures of environmental brushing PBS, consumables-washing PBS, and all reagents used in the test. In addition, some strategies should be adopted during bioinformatic analyses to remove microbial data from common contamination.^34^ We also need to take some measures to avoid false negative results caused by system factors, e.g., standard positive control throughout the procedures can be used to avoid false negatives and guarantee quantitative accuracy.

### Postanalytical problems

Postanalytical procedures covers data interpretation and results reporting. Laboratory physicians should undergo professional training on cmDNA data interpretation and reporting. Results should be analyzed and reported and combined with the patient's clinical features and other laboratory data, such as inflammation related items including clustering and counting of white blood cells, C reactive protein (CRP), and erythrocyte sedimentation rate (ESR) to put the data into perspective. Additionally, a benchmark database should be established to continuously accumulate data in the laboratory to rule out false positives caused by contamination.

## Conclusions and future perspectives

The definition of cancer microbiome research moves beyond the confines of the traditional gut microbiota into more systemic microbial research, including the intratumor microbiome and cmDNA. In fact, cmDNA reflects the overall situation of microbial burden and the results of interactions between microbes, tumors, and immunity in cancer patients. For both in-depth basic research and further clinical applications, this is a revolutionary and explosive change for this fast-growing field. In the further future, novel explorations of cmDNA will provide significant clues for malignancy prevention, control, diagnosis, and treatment to further promote the development of cancer precision medicine. With the development of detection techniques and in-depth exploration of cmDNA, TMbB, defined as a promising quantitative index of the microbiota of cancer patients, may serve as a potential biomarker in cancer diagnosis and therapy. Similar to the concept of the tumor mutation burden (TMB), TMbB is also closely related to host immunity and treatment response of tumor, while the difference is that the latter is the concept of tumor-related microbiology, and the former is the concept of tumor molecular mutation. We assume that TMbB in future research also could be divided into tumor tissue TMbB and blood TMbB. Levels of tumor tissue TMbB may be measured by the density of microbe cells in the tumor, the proportion of microbial nucleic acid *in situ* hybridization, or the proportion of microbial proteins in immunohistochemical staining. The measurement criteria for blood TMbB are mostly related to cmDNA. Furthermore, the potential roles of intratumor microbiota in oncogenesis and cancer development may provide other possible explanations for the crucial gaps of knowledge in cancer research, such as mutation genesis and evolution, regulation of cancer immunity, and other cancerous signal pathways, which may also be reflected by cmDNA. In this review, we claim that the mechanical and clinical explorations of cmDNA represent an excitingly promising direction, especially in the early-stage diagnosis of solid tumors. However, a great literature gap exists between small-sample-size observations and clinical applications targeting cancer microbiomes. In the future, the value of cmDNA in cancer precision medicine should be tested through more large-sample-size, multicentric, longitudinal studies. Overall, the necessary studies for the future are summarized as follows: (i) the source and production mechanisms of cmDNA; (ii) matched cmDNA detective techniques and methods; (iii) the roles and mechanisms of cmDNA in cancer development and invasion; and (iv) the exploration of new diagnostic methods, new therapeutic strategies, and adjuvant treatment methods targeting human systemic microbiomes. Significant achievements in these directions will provide more possibilities and options for precision medicine in oncology.
